# Gut Pathology and Responses to the Microsporidium *Nosema ceranae* in the Honey Bee *Apis mellifera*

**DOI:** 10.1371/journal.pone.0037017

**Published:** 2012-05-18

**Authors:** Claudia Dussaubat, Jean-Luc Brunet, Mariano Higes, John K. Colbourne, Jacqueline Lopez, Jeong-Hyeon Choi, Raquel Martín-Hernández, Cristina Botías, Marianne Cousin, Cynthia McDonnell, Marc Bonnet, Luc P. Belzunces, Robin F. A. Moritz, Yves Le Conte, Cédric Alaux

**Affiliations:** 1 INRA, UR 406 Abeilles et Environnement, Site Agroparc, Avignon, France; 2 Bee Pathology Laboratory, Centro Apícola Regional, JCCM, Marchamalo, Spain; 3 The Centre for Genomics and Bioinformatics, Indiana University, Bloomington, Indiana, United States of America; 4 Institut für Biologie, Martin-Luther-Universität Halle-Wittenberg, Halle (Saale), Germany; Centro de Pesquisas René Rachou, Brazil

## Abstract

The microsporidium *Nosema ceranae* is a newly prevalent parasite of the European honey bee (*Apis mellifera*). Although this parasite is presently spreading across the world into its novel host, the mechanisms by it which affects the bees and how bees respond are not well understood. We therefore performed an extensive characterization of the parasite effects at the molecular level by using genetic and biochemical tools. The transcriptome modifications at the midgut level were characterized seven days post-infection with tiling microarrays. Then we tested the bee midgut response to infection by measuring activity of antioxidant and detoxification enzymes (superoxide dismutases, glutathione peroxidases, glutathione reductase, and glutathione-S-transferase). At the gene-expression level, the bee midgut responded to *N. ceranae* infection by an increase in oxidative stress concurrent with the generation of antioxidant enzymes, defense and protective response specifically observed in the gut of mammals and insects. However, at the enzymatic level, the protective response was not confirmed, with only glutathione-S-transferase exhibiting a higher activity in infected bees. The oxidative stress was associated with a higher transcription of sugar transporter in the gut. Finally, a dramatic effect of the microsporidia infection was the inhibition of genes involved in the homeostasis and renewal of intestinal tissues (Wnt signaling pathway), a phenomenon that was confirmed at the histological level. This tissue degeneration and prevention of gut epithelium renewal may explain early bee death. In conclusion, our integrated approach not only gives new insights into the pathological effects of *N. ceranae* and the bee gut response, but also demonstrate that the honey bee gut is an interesting model system for studying host defense responses.

## Introduction

The Microsporidia constitute a group of obligate intracellular single-cell spore-forming parasites that can infect a variety of insect taxonomic orders [1], including honey bees. Indeed, honey bees, which are important for the development and maintenance of natural ecosystems and agriculture, are commonly infected by microsporidia from the genus *Nosema*. Host infection takes place after ingestion of mature spores that germinate in the midgut by polar tube extrusion and injection of the sporoplasm inside the epithelial cell cytoplasm [2]. The European (*Apis mellifera*) and the Asian (*A.cerana*) honey bees were originally parasitized by *N. apis* and *N. ceranae*, respectively [3], [4], however recent natural infections of *N. ceranae* in the European honey bee have been found across the world (see [5], [6], [7] for reviews and [8]). In its new host, *N. ceranae* is considered to cause major health problems characterized by an immune suppression [9], a degeneration of gut epithelial cells [2] and a reduction of bee lifespan [2], [10], [11], [12]. Yet, laboratory assays comparing the virulence of *N. apis* and *N. ceranae* gave contradictory results, with one study showing that *N. ceranae* is more virulent than *N. apis* [2] and a second one revealing a lack of difference in their virulence [13]. However, those divergent results might be explained by genetic differences in both the host and the parasite isolates [14], [15]. In the field, *N. ceranae* has been found to be highly virulent and a potential cause of colony collapse in Spain [6], [16] but epidemiological studies performed in the US [17], [18] and in Germany [19], [20] failed to associate this new parasite to colony losses. Those geographic differences might reflect the better adaption of *N. ceranae* to elevated temperature as compared to *N. apis* [21], [22].

Finally, if this parasite might not act on its own it can potentially interact with others stress factors since pesticide exposure increase its proliferation [23], [24] and its impact on bee health [25], [26].

Even though, lots of information has been gathered on the prevalence, development and epidemiology of this emergent parasite [5], [6], [7], little is known about how *N. ceranae* cause host damages and the mechanisms by which bees protect themselves. Yet this information is vital for designing effective diagnoses and therapeutics. Since the genome sequences of *A. mellifera* [27] is available, it seems promising to use this information to develop novel insights into the gut defense response in insects. Since ingestion is the main entry route of many pathogens, the intestinal epithelium is the first line of defense protecting the host against invasion and dissemination of pathogenic microorganisms. If the classic innate immune system plays a central role in the defense against a broad spectrum of microorganisms [28], one of the most immediate epithelial response in mammals to combat the pathogen involves the generation of antimicrobial reactive oxygen species (ROS) [29], [30]. After the ingestion of microbe-contaminated food, insects can rapidly mount an immune response involving different molecular pathways (*NF-κB*, *Toll* and *immune deficiency* pathways) [31], [32], but the production of ROS is also a key feature of this protective response [30], [33], [34]. A concurrent elimination of residual ROS is also observed to protect the host [35], since the homeostasis of redox (reduction-oxidation) balance mediated by antioxidant enzymes is essential to the host survival.

In order to investigate how honey bee gut cells respond to infection by *N. ceranae* and how the parasite affects gut epithelium, we performed a transcriptomic analysis of infected and non-infected bees using a recently developed honey bee tiling. As a complementary approach, we tested the activity of the antioxidant system, required for host protection against gut infection in *Drosophila* [35], by determining the activity of major antioxidant enzymes: the superoxide dismutases (SODs) and glutathione peroxidases (GPs). The indirect antioxidant functions carried out by enzymes such as glutathione reductase (GR), which allows the recovery of reduced glutathione from oxidized glutathione (product of glutathione reactions catalyzed by GP), and glutathione-S-transferases (GST), which catalyze the conjugation of glutathione xenobiotics [36] were also analyzed. Another enzyme that may play a key role in the maintenance of midgut homeostasis and that presents a strong activity in the midgut tissue in insects [37] is the alkaline phosphatase (AP), which, in the gut of mammals, is involved in the dephosphorylation of bacterial lipopolisaccharides (reducing their toxicity), nutrient absorption and the reduction of gut inflammation [38]. Thus, we determined the influence of *N. ceranae* infection on its activity in honey bees. Finally, to connect molecular changes induced by *N. ceranae* infection to higher-order modifications, we determined the impact of the parasite on the host midgut epithelium and mortality.

## Results and Discussion

**Figure 1:**
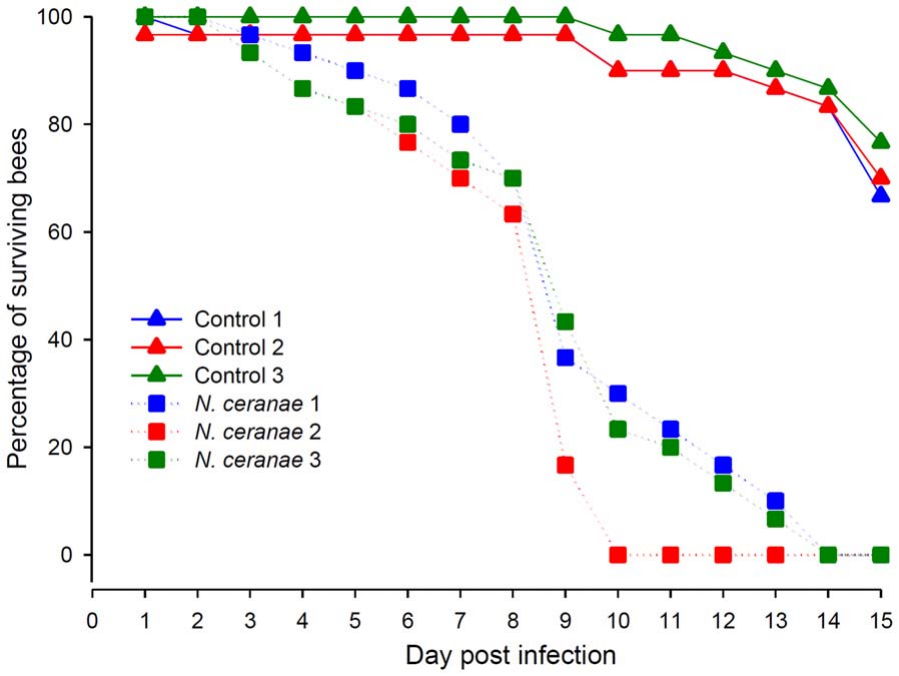
Mortality induced by *N. ceranae*. Data show the percentages of surviving bees per replicate (*n* = 3) and per day in cages composed of 30 bees each (90 bees total/treatment). Cages with *N. ceranae* infected bees achieved one-hundred percent of mortality at day 14 post infection, while in control groups mortality remained low.

The experimental infection was performed in Spain with the local subspecies of honey bee *A. m. iberiensis*. Before analysing the pathological effects of *N. ceranae* on *A. mellifera* bees, we first verified that the parasite significantly reduced the lifespan of the host, as observed by previous studies [2], [10]. The cumulative mortality rate of honey bee workers infected with *N. ceranae* was significantly higher than that of control bees (log-rank test: *p*<0.001). All infected bees died within 14 days post infection in each trial (Figure 1), showing a consistent negative effect of the parasite on bees from the different colonies used for assessing the transcriptomic and enzymatic responses to the spore infection.

### *Nosema Ceranae* Induces Oxidative Stress in the Midgut

We used a tiling microarray based on gene predictions and annotation from the honey bee genome sequencing project [27] to identify at the gut level the pathological impact of *N. ceranae* on worker bees at seven days post-infection. A total of 9,293 genes were expressed in the gut, which represents approximately 70% of the genes so far identified from the honey bee genome. Out of those genes, the transcription of 336 genes was found to be significantly altered by the proliferation of *N. ceranae* spores (see Table S1 for the list of genes). Since we analyzed bees from 3 different colonies (i.e. different genetic backgrounds) that were each headed by a multi-drone inseminated queen, those genes might represent the “general” gut pathology and/or response to the parasite.

When challenged by microorganism infections, insects can rapidly mount a potent immune response involving different molecular pathways [31]. For example, ingestion of bacteria activates the *Imd* pathway in the gut [32]. An activation of the innate immune system was therefore expected after challenging the bees with microsporidia, as it was found in drones [39]. However, no immune gene was more highly transcribed in workers after a seven-day infection. On the contrary, 2 genes (*basket* (GB16401) and *u-shaped* (GB16457)) that are involved in *Drosophila* immune responses [40], [41] were downregulated (Table S1), perhaps indicating an immune suppression by the parasites, as suggested by Antunez et al. [9]. Several studies have shown that one of the most immediate immune response of the gut involves the production of reactive oxygen species (ROS) to fight bacterial infection both in mammals [30], [42] and insects [34], [35], [43]. ROS, which are efficient antimicrobial molecules, are generally derived from oxidation-reduction process. We performed a Gene Ontology analysis to explore which functional components were affected by *N. ceranae*, and found that genes involved in “oxidation reduction” were significantly overrepresented in the gene set upregulated upon spore infection (Table 1). This increase of oxidation reduction in the gut epithelia of bees parasitized by *N. ceranae* would therefore indicate an enhanced generation of ROS in response to the infection and suggests that ROS production is a general gut immune response to microorganism infection, including microsporidia.

**Table 1:** Functional analysis of honey bee genes affected *N. ceranae* parasitism.

	GO term	# genes	*p*-value
	GO:0005886 - plasma membrane	32	1.62e-09
	GO:0030182 - neuron differentiation	23	2.48e-06
	GO:0048666 - neuron development	20	7.66e-06
	GO:0007409 - axonogenesis	14	6.03e-05
	GO:0007424 - open tracheal system development	12	1.85e-04
	GO:0007411 - axon guidance	11	1.85e-04
	GO:0001894 - tissue homeostasis	5	2.88e-04
Down	GO:0048729 - tissue morphogenesis	14	3.83e-04
	GO:0007169 - transmembrane receptor protein tyrosine kinase signaling pathway	9	3.79e-04
	GO:0050905 - neuromuscular process	4	7.85e-04
	GO:0007167 - enzyme linked receptor protein signaling pathway	10	0.001
	GO:0010647 - positive regulation of cell communication	6	0.0018
	GO:0007242 - intracellular signaling cascade	14	0.0019
	GO:0006468 - protein amino acid phosphorylation	13	0.0023
	GO:0002009 - morphogenesis of an epithelium	12	0.0026
	GO:0006979 - response to oxidative stress	5	7.14e-04
Up	GO:0055114 - oxidation reduction	11	0.0027
	IPR005829 - sugar transporter, conserved site	4	0.004

The lists of genes regulated by *N. ceranae* parasitism were analyzed for statistical enrichment of associated Gene Ontology (GO) and InterPro (IPR) terms (*p*<0.005), relative to the representation of these terms for all expressed genes.

**Figure 2:**
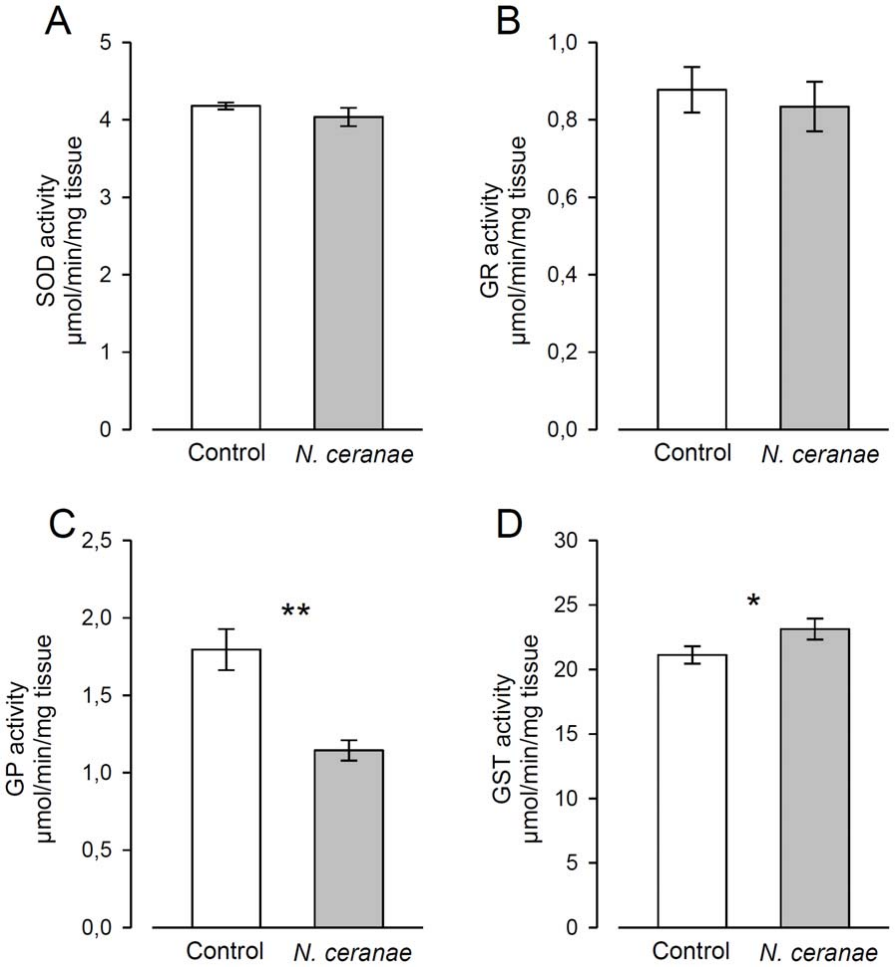
Activity of antioxidant enzymes in the midguts of bees challenged by *N. ceranae*. Differences in enzymatic activity of A) superoxide dismutase (SOD), B) glutathione reductase (GR), C) glutathione peroxydase (GP) and D) glutathione-S-transferase (GST) were estimated by a Mann-Whitney U test. Means±SE are shown for 4 pools of 3 midguts per replicate (*n* = 3 replicates, 36 bees total/treatment). * and ** denote significant differences at *p*<0.05 and *p*<0.01, respectively.

Since residual ROS can cause inflammatory disease, a balance between synthesis and elimination of ROS via antioxidants is necessary to protect the host gut [29], [34], [35], [43]. Therefore, the antioxidant system may play an essential role during gut infection. Interestingly, the functional analysis also revealed a “response to oxidative stress” in the bee gut (Table 1, see Table S2 for the list of genes associated with each Gene Ontology category), notably with the upregulation of the *catalase* (GB30227) and *glutathione peroxidase-like 2* (*Gtpx2* (GB18955)) genes, which have both antioxidant properties. A similar catalase, *the immune-regulated catalase* has been shown to be a key player of the *Drosophila* defense system during microbe infection in the gut epithelia [35]. Two cytochromes P450 were induced in the bee gut (*CYP6AS4* (GB15793) and *CYP6BC1* (GB10466)). *CYP6AS4* has been shown to metabolize quercetin contained in honey and pollen [44], but it is not known whether they might contribute to the production or elimination of ROS. We further tested whether the protective response in the bee gut increased in response to *N. ceranae* infection by measuring the activity of major antioxidant and detoxification enzymes: superoxide dismutase (SOD), glutathione peroxydase (GP), glutathione reductase (GR) and glutathione-S-transferase (GST). The activity of SOD and GR was not significantly different between control and infected bees (Mann-Whitney U tests: *p* = 0.931 and *p* = 0.558, respectively; Figure 2). Unexpectedly, the general activity of GP was significantly decreased by the spore infection (*p* = 0.002). We observed the opposite pattern for *Gtpx2* at the transcriptome level (see above). The lower GP activity may either reflect a post-translational modification or the expression level of the other GP found in the bee genome (*Gtpx1* (GB14138)) but its expression was not significantly different. The gut might also respond to a potential inhibition of GP activity upon spore infection by increasing the transcription of *Gtpx2*. Further experiments are needed to explore the GP pattern. Finally, the GST activity was induced by *N. ceranae* (*p* = 0.04 Figure 2), as recently found by Vidau et al. [26]. Different GST genes have been found to be significantly induced in the intestinal tissue after an oral bacterial infection, suggesting that GSTs might be involved in the protection of gut epithelium against pathogens [32]. The gut protection after *N. ceranae* challenge was therefore not really confirmed at the enzymatic level but a previous studies reported an increase of the total antioxidant capacity in infected queens one week post-infection [45].

### *Nosema Ceranae* Impairs Cell Signalling and Tissue Integrity in the Midgut

**Figure 3:**
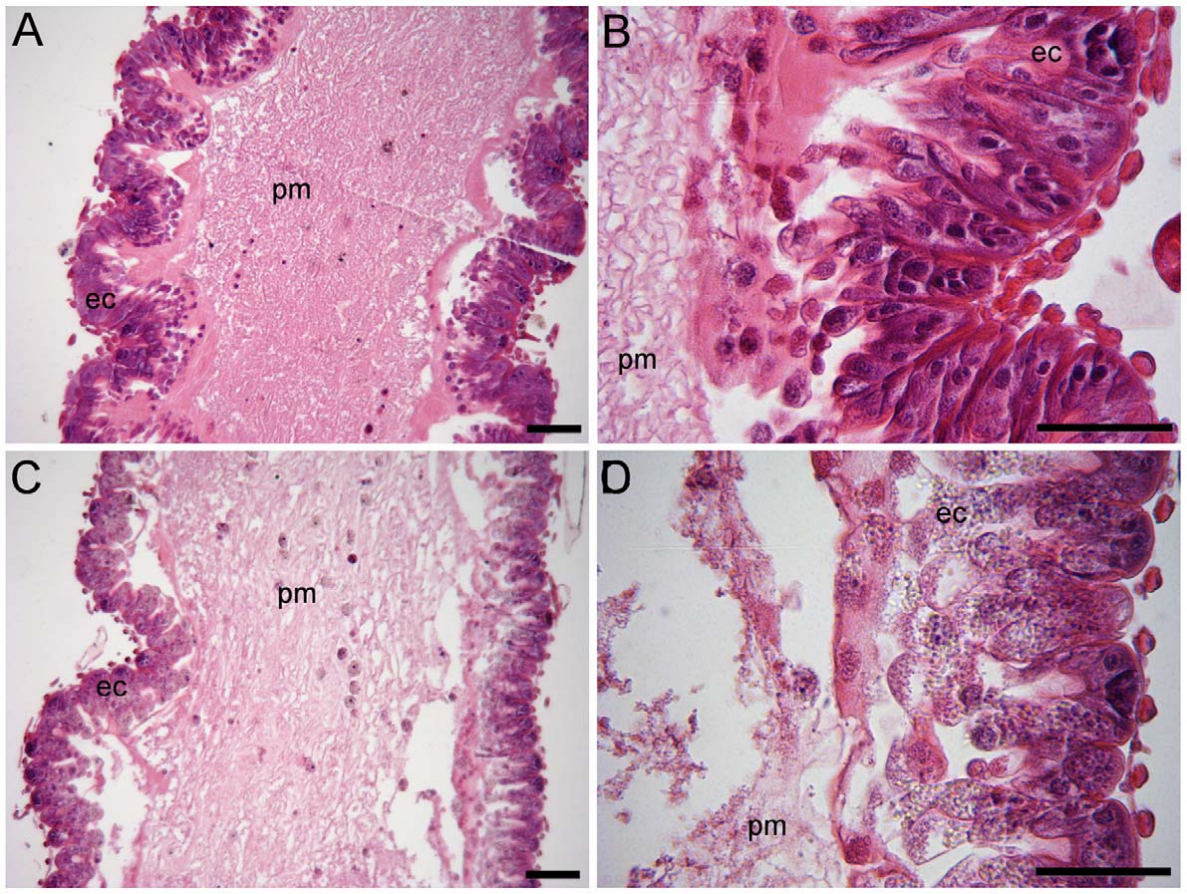
Histology of honey bee midguts 7 days post-infection. Light microscopy analysis of control (A, B) and *N. ceranae* infected midguts (C, D) stained with Hematoxylin-Eosin. In control guts, the peritrophic membrane (pm) and epithelial cells (ec) are homogenous, while in parasitized the guts peritrophic membrane and epithelial cells show signs of degeneration and lyses, respectively. Similar lesions were observed in each infected bees (*n* = 2 bees per replicate and treatment, giving *n* = 6 bees per treatment). A) and C) x100, B) and D) x400. Scale bar: 10 µm.

A significant number of genes involved in cell signaling (e.g. of GO terms: “positive regulation of cell communication”, “enzyme linked receptor protein signaling pathways”, “transmembrane receptor protein tyrosine kinase signaling pathway”) was inhibited by the parasites (Table 1). Cell-cell communication enables cells to perceive and correctly respond to their environment during tissue development and repair or the regulation of tissue homeostasis. Accordingly, the function “tissue homeostasis” was downregulated in parasitized bees, as well as biological processes implicated in “morphogenesis of an epithelium” and “open tracheal system development” (Table 1). The deregulation of “protein amino acid phosphorylation” by the parasite (Table 1) might be involved in the degeneration of the gut tissue. Since protein phosphorylation regulates many aspects of cell life, the modification of the phosphorylation states of intracellular proteins might be a cause or a consequence of the disease state [46]. These results suggest that the proliferation of *N. ceranae* caused a degeneration of the gut epithelium. Interestingly, the effect of *N. ceranae* on tissue morphogenesis and integrity at the molecular level was confirmed at the histological level (Figure 3) as in a previous study [2]. The epithelial cells of infected bees showed major signs of degeneration, which are linked to the downregulation of biological process like “positive regulation of cell communication” and “tissue homeostasis and morphogenesis” (Table 1). Gut cells are usually renewed via the multiplication and differentiation of stem cells in the basal cell layer that, once differentiated, move toward the lumen. In insects, this renewal of intestinal stem cells is controlled by the canonical Wnt signaling pathway, which includes the main downstream components *frizzled* and *armadillo* [47], [48]. Our results indicated that four main genes (*frizzled2* (GB12765), *groucho* (GB13456), *basket* (GB16401) and *armadillo* (GB12463)) from this pathway were inhibited by the parasite, suggesting that *N. ceranae* development inhibited the self-renewal of intestinal cells of the host.

This finding might be surprising, since the reaction of the gut to microorganisms involved not only the activation of the immune system, but also integrated responses controlling self-renewal and differentiation of stem cells, essential to the gut tissue homeostasis [32]. However, similar results have been found with *Encephalitozoon* microsporidia that induces a disruption of the cell cycle of the host cells [49] but without killing the host. The dramatic lifespan reduction of bees parasitized by *N. ceranae* could likely be explained by greater changes in the host cell cycle as compared to changes induced by non-lethal microsporidian species.

**Figure 4:**
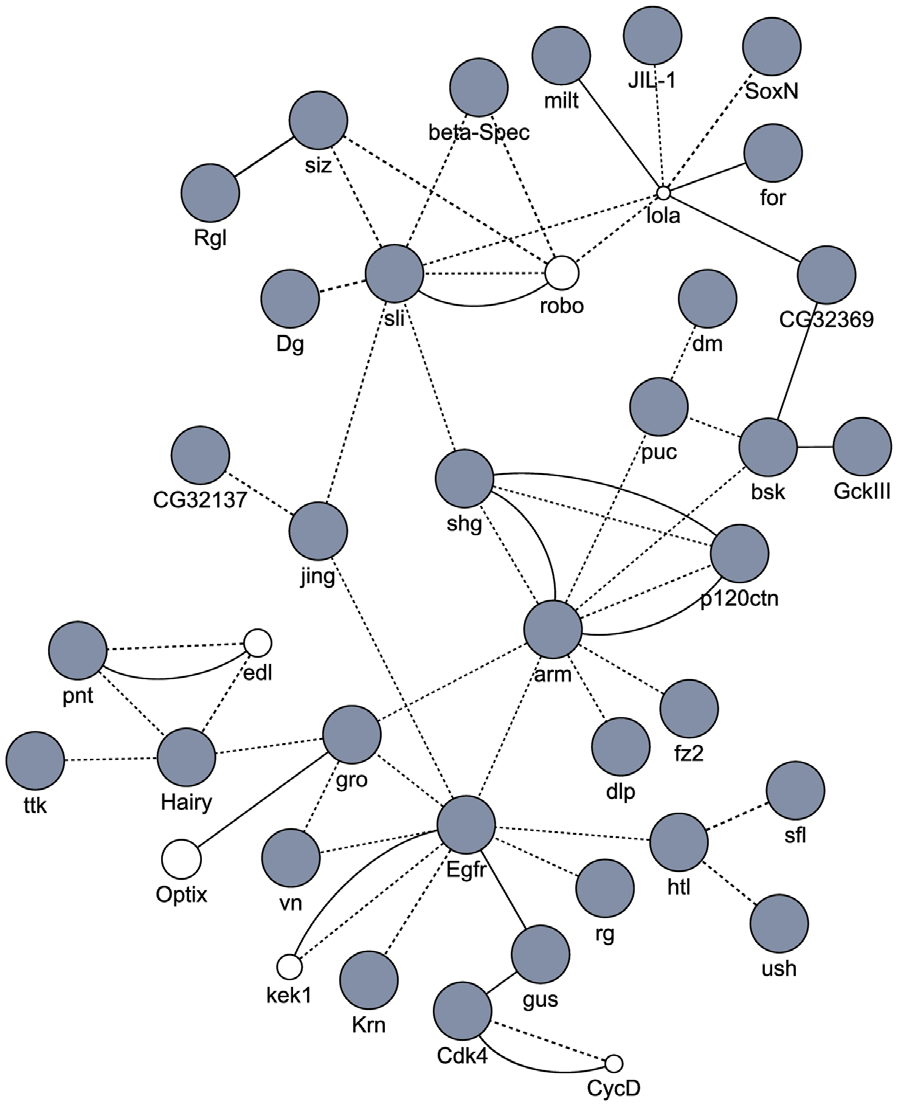
Network of genes downregulated by *Nosema* in the bee gut. The composite functional association network derived from different genomic and proteomic data sources was generated with GeneMania using the *Drosophila* orthologs of bee genes. Physical and genetic interactions between genes are indicated by dot and solid lines, respectively. Grey and white circles represent implemented genes (known genes affected by *Nosema*) and new genes predicted to be functionally associated to the known genes, respectively. The size of the predicted gene circle provides an indication of its interaction score. Except *ETS-domain lacking* (*edl*), the predicted genes *roundabout* (*robo*), *longitudinal* (*lola*), *Cyclin D* (*CycD*), *Optix*, *kekkon-1* (*kek1*) had bee orthologs: GB17658, GB12094, GB14028, GB16761 and GB17490.

We explored whether genes affected by *N. ceranae* in the honey bee gut were connected through functional networks. The network analysis was performed by testing our gene list in GeneMania, which determines whether genes are connected through physical (protein) or genetic interactions based on a large set of functional association data [50]. We found that 34 out of the 336 genes affected by *N. ceranae* were connected within a single network, characterized by 63% of physical interaction and 37% of genetic interaction (Figure 4, see Table S3 for the GB name of honey bee genes). All of those genes from this network were downregulated by the parasite, which represents 25% of the total number of downregulated genes. Interestingly, most of the genes were involved in tissue and neuron development and included the 4 main genes of the Wnt signaling pathway (*frizzled2*, *groucho*, *basket* and *armadillo*), although we cannot exclude other effects of the observed changes in expression of these genes, since a gene can be involved in different biological processes. The microsporidian might therefore induce the degeneration of the epithelial cells of the bee gut through the inhibition of this network. The functional analysis also revealed a negative impact of the parasite on neuron development and differentiation and neuromuscular process (Table 1). In insects, the enteric nervous system (ENS) of the gut is composed of interconnected ganglia and nerve plexuses that contribute to the regulation of feeding and swallowing and gut peristalsis and metabolism [51]. The impairment of the ENS is further confirmed by the inhibition of some genes involved in the circulation of Ca^2+^ and Na^+^ (*Ca2+-channel-protein-β-subunit* (GB17403), *Calcium ATPase at 60A* (GB17876), *Na pump α subunit* (GB20055), *Na^+^-driven anion exchanger 1* (GB19698)) that are important for neuromuscular transmission in insects [52], [53]. In particular mutation of the *Na pump α subunit* causes a pronounced neurodegeneration in the nervous system and reduces life span [52], [54]. Therefore, our results showed that the pathology induced by the microsporidia development is characterized by an impairment of both the epithelium and the ENS of the gut. It is also noteworthy that the corticotrophin-releasing hormone (CRH)-binding protein, which is highly conserved between insects and vertebrates [55] was upregulated in parasitized bees (Table S1). In mammals, the CRH plays an important role in mediating stress responses in the gut (i.e. increase in motility, transit, defecation, diarrhea) [56]. The upregulation of this gene might thus represent a stress response to the *N. ceranae* infection.

**Figure 5:**
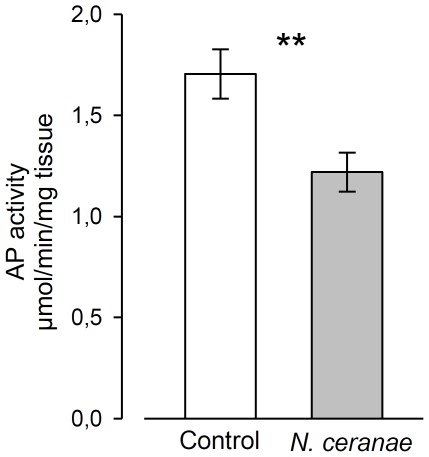
Activity of alkaline phosphatase (AP) in the midguts of bees challenged by *N. ceranae*. Means±SE are shown for 4 pools of 3 midguts per replicate (*n* = 3 replicates, 36 bees total/treatment). ** denotes significant differences at *p*<0.01 using a Mann-Whitney U test.

Finally, to further understand the pathological impact of the parasite, we measured the activity of the alkaline phosphatase (AP). Its biological role in insect gut is not well known. However, in mammals, the activity of the AP plays a pivotal role in gut health [38] since it is involved in the regulation of nutrient absorption [57], the detoxification of bacterial lipopolysaccharide [58], prevents bacterial invasion [59] and effectively reduces intestinal inflammation caused by bacteria [60]. In addition, there are numerous structural and functional homologies between insect and mammal APs [37]. Here, its activity was significantly decreased by *N. ceranae* (Figure 5, Mann-Whitney U test: *p* = 0.007), suggesting a reduction of gut protection or health. Similarly, Antunez et al. [9] found that this microsporidian induces an immune suppression in bees, which would affect the host susceptibility to others pathogens.

### *Nosema Ceranae* Increases Sugar Metabolism in the Midgut

The *α-glucosidase* gene (GB19017), which is involved in sugar metabolism, was upregulated in infected bees (Table S1). This protein hydrolyzes sucrose from the flower nectar to glucose and fructose in the hypopharyngeal glands [61], [62] suggesting an increase in sucrose breakdown in the midgut. Since carbohydrates represent the main source of energy, specialized transporter proteins are needed to transport these molecules across the plasma membrane of cells. This was confirmed by a higher transcription of genes that might be involved in the transport of trehalose (glucose-glucose), the main major carbohydrate energy storage molecule in insects (GB12932: *facilitated trehalose transporter Tret1-like*, GB13688: *trehalose transporter 1* and GB17752: *facilitated trehalose transporter Tret1-like*, Table 1 and S1). The increase of sugar metabolism is not yet clear, but previous studies clearly showed that *N. ceranae* infected bees have a higher sugar demand and consumption [11], [25], [63], [64]. This observation might be explained by the fact that microsporidia, including *N. ceranae*, are usually amitochondriate, and thus have a high dependency on host ATP [65], [66], especially for their germination. This dependence of microsporidia on host energy likely causes the increase in sucrose needs in bees, which would stimulate the expression of sugar transporter and *α-glucosidase*. However, we cannot exclude that the increase in sugar metabolism is an energy requirement for the bee gut epithelium to enhance the production of ROS in response to infection (see above).

### Expression of *Nosema Ceranae* and Other Microorganisms’ Genes

Since the tiling arrays were spotted with *N. ceranae* probes based on gene predictions and annotation from the microsporidia genome sequencing project [66] but also with gene sequences of other microorganisms, we could measure in parallel the transcriptome of the parasite and other microorganisms. In order to consider a gene to be significantly expressed we took into account the median value of all the probe fluorescence signals in a gene, but due to the weakness of the fluorescence signal for most of the probes, we did not find any microsporidia genes to be expressed in infected bees. However, the signal intensities of 174 single probes (representing 81 genes) were more highly expressed in infected bees (Table S4). Therefore, only a small fraction of the parasite genome was found to be expressed on the array, possibly representing actively transcribed and/or the most highly expressed *N. ceranae* genes at this moment of its life cycle. This is rather low in comparison to the 2,614 protein-coding genes found in the parasite genome [66], but it might be possible that RNA from spores could not be isolated due to spore wall protection, which could lead to poor hybridization and weak signal on the arrays. However, during the germination of spores, the rigid wall ruptures, allowing the polar filament to infect the cytoplasm of host cells. Alternatively, the observed signal might come from few spores that were actively germinating, since the spore count level off around 8 days post-infection [13], [67]. By conducting an Expressed Sequenced Tags (EST) survey of the microsporidian *Encephalitozoon cuniculi*, a parasite of the mosquito *Edhazardia aedis*, Gill et al. [68] also identify a small number of transcripts (133 unique genes).

We then checked whether the populations of other microorganisms were affected by *N. ceranae* infection. Only one gene of the bacteria *Paenibacillus larvae* (Plarl_010100002528, *M* = 0.48, *q* = 0.099), three genes of the fungus *Ascosphaera apis* (2 upregulated: Ascosphaera_26355, *M* = 0.52, *q* = 0.097; Ascosphaera_07711, *M* = 0.56, *q* = 0.052; 1 downregulated: Ascosphaera_15598, *M* = -0.52, *q* = 0.094) and the *Lactobacillus* sp. 16S rRNA (EF032161: *M* = 0.66, *q* = 0.05) were differentially expressed between infected and control bees. Therefore we could not really draw conclusion on changes of the microorganism population. The differential expression of bacterial RNA that is usually non-polyadenylated might be surprising at first sight, since for the microarray analysis the RNA was amplified with a reverse-transcription reaction using an initial dT-priming step. However, studies have long reported the presence or the differential expression of RNA believed to be non-polyadenylated, like rRNA, in cDNA libraries (for a review, see [69]) and cDNA microarray analysis relying on a polyadenylated tail reverse-transcription reaction (see [70], [71]), respectively, which suggest that polyadenylation has a functional role those RNA. This phenomenon could be explained by the fact that in prokaryotes and organelles both mRNAs and non-coding RNAs can be polyadenylated [72]. In those systems, polyadenylation has a functional role and is well-known to promote RNA decay by the degradosome [72], [73]. Further experiments are needed to better characterize the *N. ceranae* gene expression and the gut microorganism modifications in infected bees. Finally, we did not find any effects on virus infection but recently, a negative correlation between *N. ceranae* spore loads and deformed wing virus infection was found in the bee midgut [74].

### Conclusions

In summary, we captured the molecular events defining the bee gut response to *N. ceranae* infection, notably characterized at the gene expression level by the generation of oxidative stress. This gut immune response previously identified in *Drosophila* appears thus to be a more general phenomenon in insects. However, this mechanism does not seem to be sufficient for preventing bee mortality. Tissue degeneration and cell renewal impairment induced by infection would be two of the main factors leading to serious mortality during continuous intestine-*Nosema* infection. These pathological effects were captured late after the initial infection, while bees were dying, which gave some clues about the factors that caused the death of bees. However, future experiments will also benefit from similar analysis at the beginning of the infection or while the spores are proliferating in order to develop a complete picture of *N. ceranae* pathology. In sum, the honey bee gut is an interesting model system for studying host-pathogen relationship; and since both *N. ceranae* and *N. apis* are cross-infective across hosts in both *A. cerana* and *A. mellifera* [75], [76], cross-comparison of molecular events during infection would provide great insights into the evolution of host-microsporidia interactions.

## Materials and Methods

### *Nosema Ceranae* Infection and Bee Rearing

In order to determine the transcriptomic and enzymatic changes induced by *N. ceranae* in the midgut of honey bees, we performed experimental infections. They were performed at the Regional Apicultural Center in Marchamalo, Central Spain, with *A. m. iberiensis*. Frames of capped brood were obtained from healthy colonies located 20 km away from CAR to provide a supply of newly emerged honey bees free of *Nosema* for all trials (*Nosema*-free honey bees confirmed by PCR following method from Martín-Hernández et al. [77]). After emergence bees were kept in an incubator at 33°C (±1°C) (Memmert Mod. IPP500) until they were 7-day old. Then, bees were starved for 2 hours and then anesthetized with CO_2_ to facilitate individual feeding with 2 µl of 50% sucrose solution containing 100,000 fresh spores of *N. ceranae*. This dose, currently found in naturally infected bees [78] and ten times higher than the minimal infectious dose required to infect all the bees [13], was chosen to guarantee a successful infection of each bee and reduce the variability in infection between bees. Purified spores of *N. ceranae* were obtained from experimentally infected honey bees and the spore concentration was calculated using haemocytometer chamber [2]. Controls were fed only with the sucrose solution. After that, bees were introduced in cages and reared in two separated incubators at 33°C (±1°C), one containing *N. ceranae* infected bees and the other one containing non-infected bees in order to avoid cross contamination. They were fed *ad libitum* with water, a solution of sucrose (50% w/w in water) and 2% Promotor L (Calier Laboratory), a commercial mixture of amino acids and vitamins.

Three experiments consisting of control and *N. ceranae*-infected groups were performed to assay the effect of the microsporidia on mortality (30 bees per cage), transcriptomic (20 bees) and enzymatic activity (50 bees). Each experiment was repeated 3 times (one repetition corresponding to one cage) on 3 colonies randomly distributed within the apiary in order to avoid any bias due to colony genetics and to analyze the “general” host/pathogen interactions. In the mortality assay, dead bees were counted daily and the mortality experiment ended when all bees infected with *N. ceranae* were dead; results were compared using a log-rank test. For the transcriptome and enzymatic activity analysis of the nosemosis type C, bees were flash frozen in liquid nitrogen and store at −80°C seven days post-infection, just before the infection level (spore count) reaches a plateau [13], [67]. At this time *N. ceranae* infection is largely developed in the midgut, while bees are still alive [2]. A few days later, bees died *en masse* (Figure 1).

### RNA Labeling, Array Hybridization and Statistical Analysis

Gene expression was analyzed in honey bee midguts. Twelve bees per group were treated with RNA later Ice (Ambion) following kit instructions and midguts were dissected on dry ice. Pools of four midguts were homogenized in Trizol (Invitrogen). Whole infected midguts containing *Nosema ceranae* or control midguts were homogenized with a hand motor-driven grinder (Kondes) and RNA extraction was carried out as indicated in the Qiagen RNeasy kit (Qiagen) for total RNA with on-column DNase I treatment (Qiagen). For each group, RNA isolated from three samples was equally pooled giving a larger pool of 12 midguts that was used for microarray analysis. Three pools of *Nosema* infected bees were then directly compared to three control pools (one pool of each per colony) using a set of four custom NimbleGen HD2 tiling microarrays containing sequences from the following draft genomes (Table S5): the honey bee *A. mellifera* version Amel_4.0, NCBI AADG00000000 interrogating 9,295 annotated gene models and 12,581 transcripts [27]; the bacterium *Paenibacillus larvae*, NCBI AARF00000000 interrogating 4,718 of 5,019 annotated genes [79]; the filamentous fungus *Ascosphaera apis*, NCBI AARF00000000 [79]; the microsporidian *Nosema ceranae*, NCBI ACOL00000000 interrogating 2,295 of 2,678 annotated genes [66]; and 10 viruses including *Varroa destructor* virus 1, NCBI AY251269 [80], Deformed wing virus, NCBI NC_004830 [81], Sacbrood virus, NCBI NC_002066 [82], *Kakugo* virus, NCBI NC_005876 [83], Chronic bee paralysis virus RNA 1, NCBI NC_010711 [84], Chronic bee paralysis virus RNA 2, NCBI NC_010712 [84], Kashmir bee virus, NCBI NC_004807 [85], Black queen cell virus, NCBI NC_003784 [86], Israel acute paralysis virus of bees, NCBI NC_009025 [87], and Acute bee paralysis virus, NCBI NC_002548 [88] and 16 s ribosomal RNA sequences from different bacteria, including probiotics (see Table S5 for a list). Note that only 1,540 out of 9,244 unmapped *A. mellifera* sequences are represented on the microarrays, those being the first 1540 sequences of the “GroupUn” set from assembly 4.0. The probes on the microarrays range in size between 50 to 60 bp, and are tiled across the unique sequences of genomes at median distances of 25 bp (mean = 33 bp); only 76,315 probes are tiled at distances >100 bp and 4,285 probes are tiled at distances >1000 bp. The microarray platform is deposited at NCBI GEO under the MIAME-compliant accessions GPL11147, GPL11148, GPL11149, GPL11150.

Total RNA concentration and integrity was determined using the Nanodrop ND-1000 (ThermoScientific) and the Bioanalyzer 2100 with the RNA6000 Nano Kit (Agilent Technologies), respectively. Total RNA was primed with oligo-dT-T7 primer and converted to amplified RNA using MessageAmpII aRNA Amplification Kit (Ambion) according to the manufacturer’s recommendations. Then, amplified RNA was primed with Random hexamer Primer (Promega) and converted to double stranded (ds) cDNA using Double-stranded cDNA Synthesis kit (Invitrogen). After treating samples with RNaseA, ds cDNA was purified with ChargeSwitch PCR Clean-Up Kit (Invitrogen). Next, ds cDNA (1000 ng) was labeled using NimbleGen Dual Color Labeling Kit (Roche NimbleGen) in triplicate following the kit’s instructions to produce enough labeled product for 3×2.1 M microarray chips. Hybridization and post-hybridization washing was performed using Hybridization Kit (Roche NimbleGen) and Wash Buffer Kit (Roche NimbleGen) according to manufacturer’s recommendations.

Arrays were scanned using an Axon GenePix 4200A Professional scanner (Molecular Devices, Sunnyvale, CA), 5 micron resolution and images analyzed with NimbleScan 2.5 Software (Roche NimbleGen) and raw signal intensities extracted as PAIR files. First, replicate arrays were quantile normalized [89] and to each probe the median value of the replicate probe values was assigned. The fluorescence signal of random probes, designed to reflect the genome nucleotide composition by Markov modeling, was used to determine a false positive rate threshold. Probes were considered positive if their fluorescence signal was higher than the 99th percentile of the fluorescence signal of the random probes. The data analysis to measure differential expression of genes was performed using the statistical software package R (R-Project 2009) [90] and Bioconductor [91] with additions and modifications. The signal distributions across chips, samples and replicates were adjusted to be equal according to the mean fluorescence of the random probes on each array. All probes including random probes were quantile-normalized across replicates. Expression-level scores were assigned for each predicted gene based on the median log2 fluorescence over background intensity of probes falling within the exon boundaries. We calculated the per-gene, per-treatment differential expression (DE) levels with LIMMA R package [92] producing M, A expression estimates, t-statistic, probability, and probability adjusted for multiple testing by using the Benjamini-Hochberg method. The data are deposited at NCBI GEO under the MIAME-compliant accession GSE25455.

### Functional Analysis

We tested whether molecular functions or biological processes from the list of genes differentially expressed after *N. ceranae* parasitism were represented by larger numbers of genes than expected by random. *Drosophila melanogaster* orthologs were identified by reciprocal best BLASTX match to honey bee genes, and Gene Ontology (GO) terms were assigned based on annotation of *Drosophila* genes. Then, DAVID 6.7 bioinformatic resources was used to identify overrepresented terms (molecular function and biological process) [93].

### Network Analysis

Network analysis was performed by analyzing all differentially expressed genes in GeneMania, which uses a large set of functional association data including protein and genetic interactions [50]. The algorithms underlying the bioinformatic tools consists of two parts: 1) a linear regression-based algorithm that calculates a single composite functional association network from multiple data sources and 2) a propagation algorithm for predicting gene function given the composite functional association network. This principle enable to predict new genes functionally associated with known genes (see [50] for details). In GeneMania, *Drosophila* was selected and the physical (protein-protein interaction data) and genetic (genetic interaction data) network options were enabled. See GeneMania website for detailed instructions. Cytoscape (version 2.7.0) was used to display the networks.

### Verification by Reverse-transcriptase PCR

Confirmation of some of the results obtained from tiling array analysis was performed with quantitative PCR (qPCR) on selected genes affected by *N. ceranae* parasitism. The qPCR analysis was carried out with 6 RNA pools of 4 bees per treatment, which included the RNA stocks used for tiling array analysis. For cDNA synthesis, 500 ng of RNA per sample was reverse-transcribed using the SuperScript III kit (Invitrogen, France). The transcript abundance was measured for the transcription factor *Hairy* [94]), *Slit*, which is involved in neuron differentiation [95] and *α-glucosidase* [96] with an Mx3000P QPCR Systems (Agilent) and the SYBR green detection method (Agilent). qPCR values of the selected genes were normalized to the housekeeping gene *eIF3-S8*, that did not vary in expression levels on the tiling array (*q*-value = 0.99). Relative expression was calculated by raising 2 to the power of the difference in Ct values. Primer sequences (5′ to 3′) were: *Hairy* (GB14857) forward: CCAGCGCGACACTCGAAGCT, reverse: AAACCTGCCAACCTCGCCGG; *Slit* (GB19929) forward: AGGCATCACGCGGAGAACGC, reverse: CGGCGGGCAACCGAGTATCC; *α-glucosidase* (GB19017) forward: TTGCTGCCAGGTGTTGCCGT, reverse: TTGGAATGGCGTTCTCGCGGG; *eIF3-S8* (GB12747) forward: TGAGTGTCTGCTATGGATTGC, reverse: TCGCGGCTCGTGGTA from [97]. Results were consistent with the microarray results (Figure S1).

### Enzyme Activity Analysis

The enzymatic activity of superoxide dismutase, glutathione reductase, glutathione peroxydase, glutathione-S-transferase and alkaline phosphatase was measured at day 7 post infection. Measures were carried out on honey bee midguts that were dissected and stored at -80°C until analysis. All analysis were done with 3 replicates/treatment and using 4 pools of 3 midguts per replicate. The effect of *N. ceranae* parasitism on enzyme activities was assayed using Mann-Whitney U tests.

#### Enzyme extraction

Samples were homogenized at 4°C with a TissuLyser (Qiagen) (5×10 s at 30 Hz) in phosphate buffer pH 7.4 (40 mM sodium phosphate, 10 mM NaCl, 1% (w/v) Triton X-100, containing a mixture of 2 mg/ml of antipain, leupeptin and pepstatin A, 25 units/ml of aprotinin and 0.1 mg/ml of trypsin inhibitor) to make a 10% (w/v) extract. The homogenates were then centrifuged at 15,000 g for 20 min at 4°C and resulting supernatants were used for further analysis of enzymatic activities and protein contents.

#### Superoxide dismutase

SOD activity was measured indirectly as the rate of reduction of nitroblue tetrazolium when superoxide anion radical was generated during oxidation of xanthine by xanthine oxidase as described by Boldyrev et al. [98]. The reaction mixture contained 50 mM sodium carbonate dissolved in 50 mM phosphate K/Na buffer (pH 7.8 at 25°C), 0.1 mM EDTA, 0.1 mM xanthine, 0.0833 U/ml xanthine oxidase and 0.025 mM nitroblue tetrazolium. The enzyme activity was assayed at 560 nm for 5 min at 25°C.

#### Glutathione reductase

Glutathione reductase activity was measured in a reaction mixture containing 50 mM Phosphate Na/K buffer (pH 7.4 at 25°C), 1 mM EDTA, 0.16 mM NADPH and 0.8 mM oxidized glutathione (substrate) as described by Boldyrev et al. [98]. The enzyme activity was assayed at 340 nm for 5 min at 25°C.

#### Glutathione peroxidase

Glutathione peroxidase activity was monitored as described by Boldyrev et al. [98] at 340 nm in a reaction mixture containing 50 mM phosphate K/Na buffer (pH 7.8), 1 mM EDTA, 0.12 mM NADPH, 0.85 mM reduced glutathione as a substrate, 0.5 unit/ml glutathione reductase and 0.2 mM tert-butyl hydroperoxide. The reaction was followed for 10 min at 25°C.

#### Glutathione-S-transferase

Glutathione-S-transferase activity was monitored by following the conjugation of reduced glutathione to 1-chloro-2,4-dinitrobenzene using a method adapted from Habig et al. [99]. GST activity was measured by adding enzymatic extract to the reaction mixture containing 1 mM EDTA, 2.5 mM reduced glutathione, 1 mM 1-chloro-2,4-dinitrobenzene and 100 mM Na/K-phosphate pH 7.4. GST activity was followed spectrophotometrically at 340 nm during 5 min at 25°C.

#### Alkaline phosphatase

AP activity was monitored at 410 nm in a medium containing 20 µM of MgCl2, 2 mM of *p*-nitrophenyl phosphate as a substrate and 100 mM Tris-HCl pH 8.5 [100]. The enzyme activity was assayed for 5 min at 25°C.

### Histology

We also checked the tissue integrity of 2 bees per replicate and treatment, giving a total of *n* = 6 bees per treatment. Midguts from infected and non-infected honey bees were fixed in formalin-acetic-alcohol 70° (5/5/90) for 24 hrs at 5–6°C, then rinsed 3×1 hr in water, dehydrated in ethanol and stored in butanol. Tissues were embedded in paraffin (Histowax, Histolab - Products AB) and sectioned at 7 µm thickness with a microtome (Leitz 1512). The following sections were stained with Hematoxylin-Eosin and photographed using a light microscope VWR (TR500 High End Tri).

## Supporting Information

Figure S1 **Validation of microarray results with qPCRs.** Expression level of 3 genes chosen among the set of genes differentially transcribed between control (black bars) and *N. ceranae*-infected bees (white bars). Data are normalized to expression levels of *eIF3-S8.* Means±SE are shown for 6 pools of 4 bees per treatment (24 bees total/treatment). Significant differences were determined using Mann-Whitney U tests (*Hairy*: *p* = 0.041, *Slit*: *p* = 0.002, *α-glucosidase*: *p* = 0.004). * and ** denote significant differences at *p*<0.05 and *p*<0.01, respectively.
(TIFF)

Table S1 List of honey bee genes differentially expressed after *N. ceranae* parasitism. The *M*-value- log2 fold change and the *q*-value are given for each gene. Corresponding honey bee genes and *Drosophila* orthologs are shown.
(XLSX)

Table S2 List of genes associated with each Gene Ontology category (see Table 1). GB accession number, fly orthologs and M-and q-values are shown.
(XLSX)

Table S3 List of genes integrated within the gene network downregulated by *N. ceranae* parasitism (see Figure 4). GB accession number, fly orthologs and M-and q-values are shown.
(XLSX)

Table S4 List of *N. ceranae* probes significantly expressed. The *M*-value- log2 fold change and the *q*-value are given for each probe. Pfam and BLAST data on *Encephalitozoon cuniculi* genome originated from Cornman et al. [66].
(XLSX)

Table S5 Tiling array summary.
(XLSX)
