# Supplementary figures and images for: Gut Pathology and Responses to the Microsporidium Nosema ceranae in the Honey Bee Apis mellifera
Source: PLoS One. 2012 May 18;7(5):e37017. doi: 10.1371/journal.pone.0037017 (PMC3356400; doi:10.1371/journal.pone.0037017)

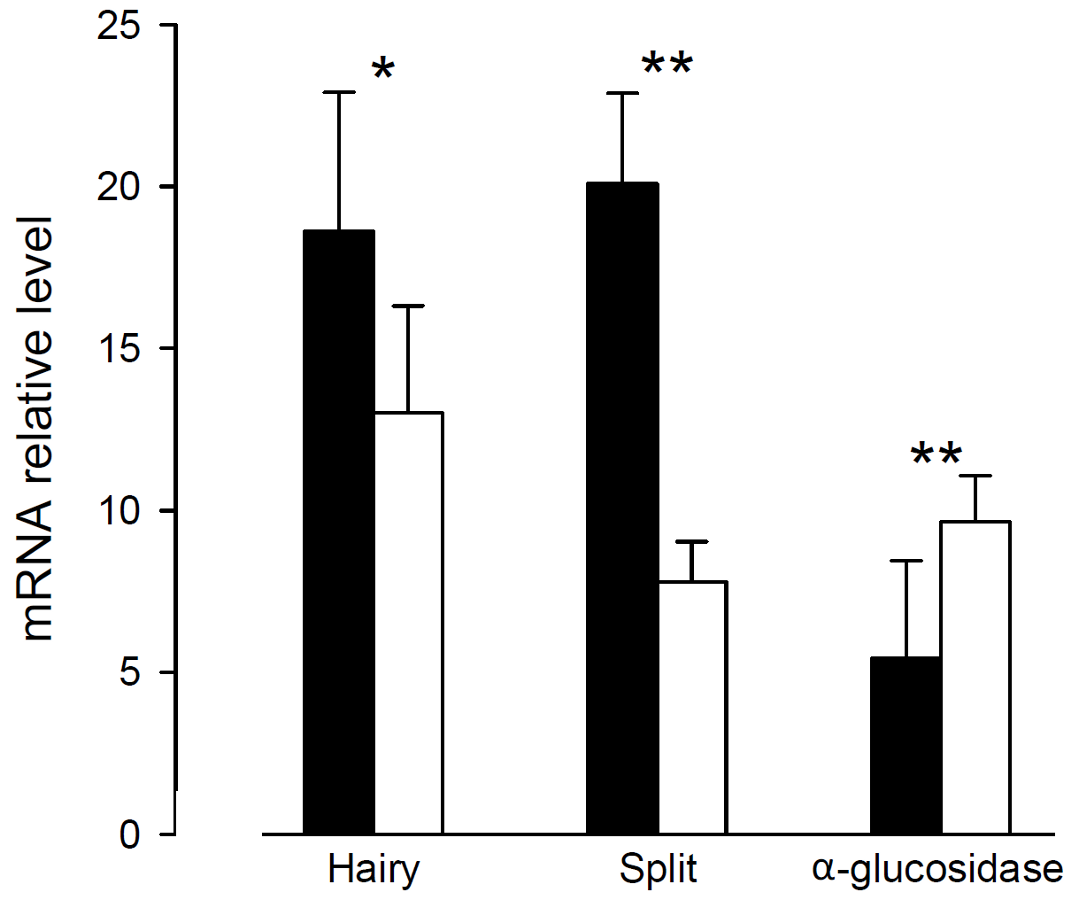

Supplement: Figure S1 — Validation of microarray results with qPCRs. Expression level of 3 genes chosen among the set of genes differentially transcribed between control (black bars) and N. ceranae-infected bees (white bars). Data are normalized to expression levels of eIF3-S8. Means±SE are shown for 6 pools of 4 bees per treatment (24 bees total/treatment). Significant differences were determined using Mann-Whitney U tests (Hairy: p = 0.041, Slit: p = 0.002, α-glucosidase: p = 0.004). * and ** denote significant differences at p<0.05 and p<0.01, respectively. (TIFF) [file pone.0037017.s001.tiff]
